# Nucleolus and chromatin

**DOI:** 10.1007/s00418-018-1696-3

**Published:** 2018-07-25

**Authors:** Christian Schöfer, Klara Weipoltshammer

**Affiliations:** Division of Cell and Developmental Biology, Center for Anatomy and Cell Biology, Medical University of Vienna, Schwarzspanierstr. 17, 1090 Vienna, Austria

**Keywords:** NOR, rDNA, rRNA, Transcription, Epigenetics, NAD

## Abstract

The nucleolus as site of ribosome biogenesis holds a pivotal role in cell metabolism. It is composed of ribosomal DNA (rDNA), which is present as tandem arrays located in nucleolus organizer regions (NORs). In interphase cells, rDNA can be found inside and adjacent to nucleoli and the location is indicative for transcriptional activity of ribosomal genes—inactive rDNA (outside) versus active one (inside). Moreover, the nucleolus itself acts as a spatial organizer of non-nucleolar chromatin. Microscopy-based approaches offer the possibility to explore the spatially distinct localization of the different DNA populations in relation to the nucleolar structure. Recent technical developments in microscopy and preparatory methods may further our understanding of the functional architecture of nucleoli. This review will attempt to summarize the current understanding of mammalian nucleolar chromatin organization as seen from a microscopist’s perspective.

## Introduction

The nucleolus is the site of the highest RNA synthesis rate in cell nuclei giving rise to more than 60% of the entire RNA pool. Compared to highly transcribed nucleoplasmic gene foci, the nucleolus can be considered as particularly active transcription hub or transcription factory. While there are common principles as to the spatial organization of nucleoplasmic and nucleolar transcription (Weipoltshammer and Schöfer 2016) differences between the constituents of both nuclear transcription factories exist. Ribosomal RNA (rRNA) is transcribed by RNA polymerase I (Pol I), which depends on a set of basal transcription co-factors including selective factor 1 (SL1), and the upstream-binding factor (UBF). Part of the transcription machinery remains bound to promoter regions of ribosomal DNA (rDNA) throughout the cell cycle yet in a de-phosphorylated, transcriptionally inactive form. Silencing of rRNA genes relies on epigenetic factors such as the NoRC complex (Santoro et al. 2002). Epigenetic regulation of rRNA transcription is an essential target for the integration of cell autonomous and environmental cues leading to adaptation of the cytoplasmic ribosome equipment available for protein synthesis. In addition, mechanisms involved in malignant transformation converge on the nucleolus to satisfy the increased demand for ribosomes of cancer cells, e.g., c-Myc increases, whereas p53 reduces rRNA transcription. This discovery revived the interest in nucleolar regulation in pathology and led to the development of therapeutics specifically targeting nucleolar transcription (Drygin et al. 2011; van Sluis and McStay 2014). Given the importance of epigenetic regulation of rRNA transcription, it is pivotal to understand the epigenetic makeup of ribosomal DNA. Despite decades of efforts, there exist still numerous open questions as to the structure and composition of ribosomal chromatin and its dynamic regulation. Reason might be the difficulties to directly address rRNA genes with molecular tools due to their highly repetitive nature best exemplified by the still lacking DNA sequence coverage of the rDNA loci in the human genome databases. In comparison to molecular studies, microscopy-based approaches have the advantage that ribosomal genes can be addressed as to their expression level and to their position within the nucleolus at the same time. Thus, it is possible to investigate the spatial distribution of differently expressing ribosomal genes and, in addition, to explore their behavior under transcriptional activation or silencing. Furthermore, recent advances in microscopy allow studying the functional nucleolar architecture with unprecedented detail.

## A short history of nucleoli and chromatin

**Fig. 1:**
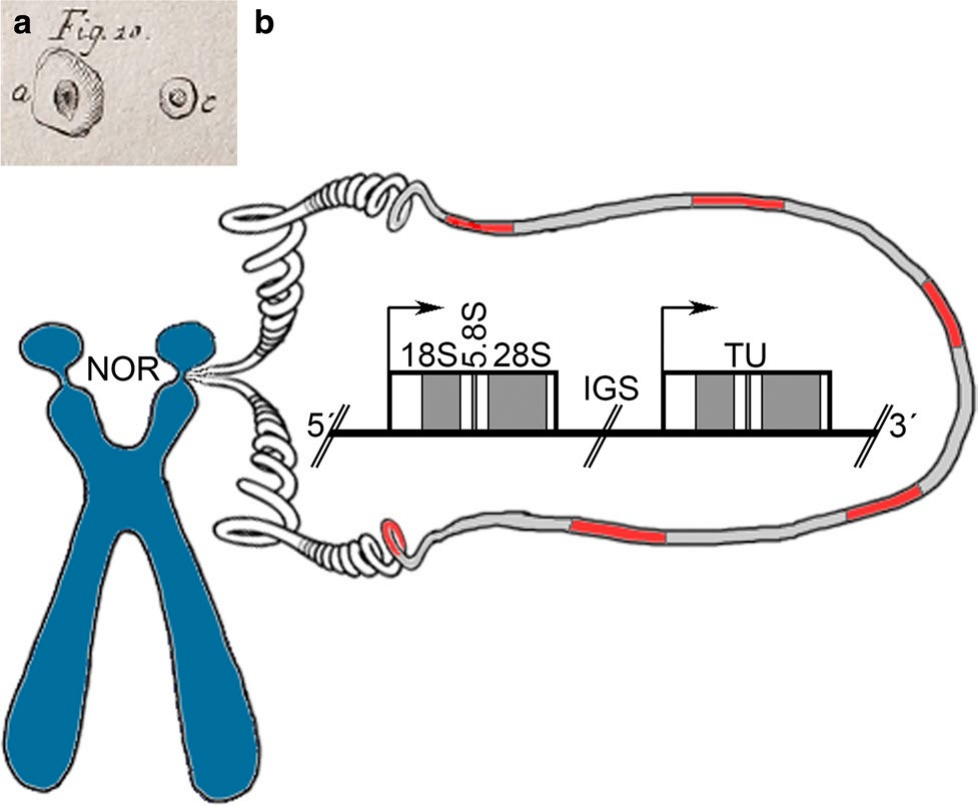
**a** One of the first depictions of what was later coined nucleolus in an eel skin cell (Fig. 10a) in size comparison to a fish erythrocyte (Fig. 10c); taken from Fontana (1781). **b** Schematic representation of nucleolar organizer regions (NOR) on a human acrocentric chromosome, the rDNA array, and the ribosomal gene organization with mature rRNAs indicated; *TU* transcription unit (rRNA gene), *IGS* intergenic spacer sequence; DNA loop: red: TU, gray: IGS

The structure which we know as nucleolus was the first nuclear body independently described by microscopists at the late eighteenth/early nineteenth century in plant and animal cells [Fig. 1a; (Fontana 1781; Valentin 1836; Wagner 1835)] and was identified as regular constituent of the nucleus (Schleiden 1838; Schwann 1839). Functional attributions remained highly speculative these days; for example, a pivotal role for nucleoli in generating new cells was proposed (Schleiden 1838). Nucleoli were found to vary in size and shape in different tissues, and to contain distinct internal structures (Montgomery 1899). Much later, the advent of electron microscopy revealed a complex tri-partite internal composition [see below, e.g., Bernhard et al. (1952), Smetana and Busch (1964), and Yasuzumi et al. (1958)].

Despite the discoveries of nucleic acid (Miescher 1871), of chromosomes, chromatin, and histones (Boveri 1909; Flemming 1880; Kossel 1884; Rabl 1885; Waldeyer 1888) by the end of the nineteenth century, it was only in the early 1930s that a link between the morphological structure of nucleoli and chromosomes was established, when it was found that nucleoli associate with a specific chromosomal locus (Heitz 1931; McClintock 1934) termed nucleolus organizer region [NOR; (McClintock 1934)]. In the 1960s, it was settled that the nucleolus houses both DNA and RNA, the latter of which being transcribed there (Fakan and Bernhard 1971; Granboulan and Granboulan 1964, 1965). In situ hybridization experiments demonstrated that these represent ribosomal RNA and DNA (Brown and Gurdon 1964; Wallace and Birnstiel 1966), and that the nucleolus is thus the site of rRNA transcription. Miller and Beatty (1969) were able to visualize rRNA transcription in spread preparations as the well-known Christmas trees. Refinement of the functional architecture was achieved by applying cytochemical methods to stain DNA (osmium-ammine), RNA (EDTA regression and terbium) and histones (acrolein-silver nitrate) (Bernhard 1969; Biggiogera and Fakan 1998; Cogliati and Gautier 1973; Derenzini et al. 1982, 1985) as well as immunogold methods (Baschong et al. 1985), which enhanced specificity in the detection of epitopes at the ultrastructural level. Since then, major constituents of nucleoli and factors that control nucleolar transcription were identified. In particular, it became clear that epigenetic factors play important roles in rRNA transcription regulation [reviewed in Grummt and Langst (2013)].

Nevertheless, despite intensive research efforts, the precise structure function relation in nucleoli is still not fully understood. One reason for the relatively slow progress might lie in the still limited tools available to study repetitive genomic elements such as rDNA arrays at the molecular level. These tools are now emerging allowing a combined approach employing microscope-based and molecular techniques.

## Human rDNA, nucleolar morphology, and rRNA transcription

### Structure of the human rDNA

In human cells, rDNA is located on the p arms of the ten acrocentric, NOR-bearing chromosomes [13–15, 21, and 22; (Henderson et al. 1972)]. Within each NOR, rDNA is arranged as clusters of repetitive elements (Sylvester et al. 1986) composed of rRNA genes (also named transcription units) separated by intergenic spacer sequences (IGS; Fig. 1b). The genes give rise to the 47S nascent transcripts (Dundr and Olson 1998), which consist of the 18S, 5.8S, and 28S rRNAs, 5′, 3′ external transcribed spacer sequences and of two internal transcribed spacers flanking 5.8S rRNA. External and internal transcribed spacer sequences separate the mature rRNA sequences and are excised in a sequence of steps that start co-transcriptionally. The three mature rRNAs together with 5S rRNA build the functional core of the two ribosomal sub-units. 5S rRNA is transcribed by Pol III outside nucleoli and is, subsequently, imported. The IGS is an important factor for the regulation of transcription. It harbors enhancer elements and gives rise to RNA molecules that either recruit factors involved in transcriptional control or alter the chromatin state and thereby may have a function in the spatial organization of the rDNA array.

### Structure of rDNA arrays

The total number of rRNA genes per diploid human genome was estimated to be in the range of approximately 400 copies (Gibbons et al. 2014; Schmickel 1973). However, the repetitive rDNA clusters are genomic hotspots for recombination events, and thus, the total number as well as the distribution of genes over the ten NORs vary dramatically between individuals (Gibbons et al. 2014; Stults et al. 2008). The copy-number variation (CNV) of the rDNA arrays seems to be concerted with the copy number of the extra-nucleolar 5S rDNA array located at chromosome 1 in humans [cCNV; (Gibbons et al. 2015)], which consists of 50–300 repeats (Stults et al. 2008). Only a fraction of rRNA genes is actively transcribed at each time point. This suggests a well-buffered genomic equipment of rRNA gene dosage able to respond to a broad range of metabolic demands.

The number of active genes is species- and tissue-specific, and for human fibroblasts, a mean number of 115 actively transcribed genes per cell has been estimated (Haaf et al. 1991).In addition to the total number of rRNA genes, each rDNA array is highly polymorphic and can undergo dramatic modifications. For example, fiber-FISH experiments demonstrated length variations within rDNA fibers (Schöfer et al. 1998). Inversions of arrays, palindromic structures, and the reversal of the head-to-tail orientation of ribosomal genes have consequences for the number of potentially active genes available at a particular NOR (Caburet et al. 2005). In yeast rDNA, array shortening has been correlated with cellular senescence. The yeast histone deacetylase Sir2 guards against this aging process (Saka et al. 2013). Loss of the mammalian Sir2-homolog SIRT7 induces rDNA array instability followed by cellular senescence (Paredes et al. 2018). These findings support a theory, whereby rDNA instability is a major driver of cellular aging (Kobayashi 2008).

Moreover, amplification as well as loss of rRNA genes occur in malignant cells. Indeed, both of these conditions were identified in different human cancer types (Udugama et al. 2018; Wang and Lemos 2017; Xu et al. 2017). Increased rRNA transcription is a ubiquitous and well-known hallmark of cancer cells and the resulting increased nucleolar sizes were used as diagnostic marker by pathologists. It is somewhat surprising that increased rRNA production can be achieved even if the rDNA copy number is reduced (Wang and Lemos 2017), yet it indicates equipment with more than sufficient numbers of rRNA genes in normal cells. Amplification of 5S rRNA has been proposed to occur concerted with 47S rDNA amplification, whereas loss of 47S rDNA may occur as consequence of a transcription–replication conflict in rapidly replicating cancer cells (Wang and Lemos 2017).

Heterogeneity with respect to activity occurs between and within NORs. Schlesinger et al. (2009) found that one parental copy of each NOR is repressed in any individual cell, which is maintained once established. Another report showed the co-existence of different epialleles of DNA methylation patterns, i.e., co-existence of active and inactive rRNA genes within individual NORs (Zillner et al. 2015).

### NORs and transcriptional requirements

Nucleoli are sensitive to changes in metabolic requirements of the cell and regulate ribosome biogenesis accordingly. Two mechanisms have been reported in yeast to adapt rRNA transcription rate. In mutant yeast cells equipped with a minimal number of rDNA, it was found that cells respond to increased demands of protein synthesis with a higher occupancy of Pol I molecules per active rRNA gene unit (French et al. 2003). However, in normal yeast cells, it seems that no significant changes exist in Pol I occupancy per gene during growth retardation (Johnson et al. 2013). In mammalian cells, the number of active genes remain constant under stable metabolic conditions and during cell cycle (Conconi et al. 1989; Haaf et al. 1991). Under conditions of changing demand for ribosomes, new rRNA genes are recruited for transcription or, conversely, are silenced. In the latter case, they may become either permanently silenced or they can be converted into a poised state (see below), which is competent of transcriptional activation. It was shown that differentiated cells have a higher amount of rRNA genes with a poised signature (Xie et al. 2012).

During the cell cycle, rDNA is replicated in a biphasic manner with actively transcribed genes replicating early in S phase, whereas silent are late replicating in S phase (Berger et al. 1997; Li et al. 2005). Recently, it was reported that correlation of replication timing and Pol I occupancy suggests that a considerable subset of ribosomal genes remain transcriptionally silent from mid S phase to mitosis, but become again active in the postmitotic daughter cells (Smirnov et al. 2016).

### Nucleolar morphology

**Fig. 2:**
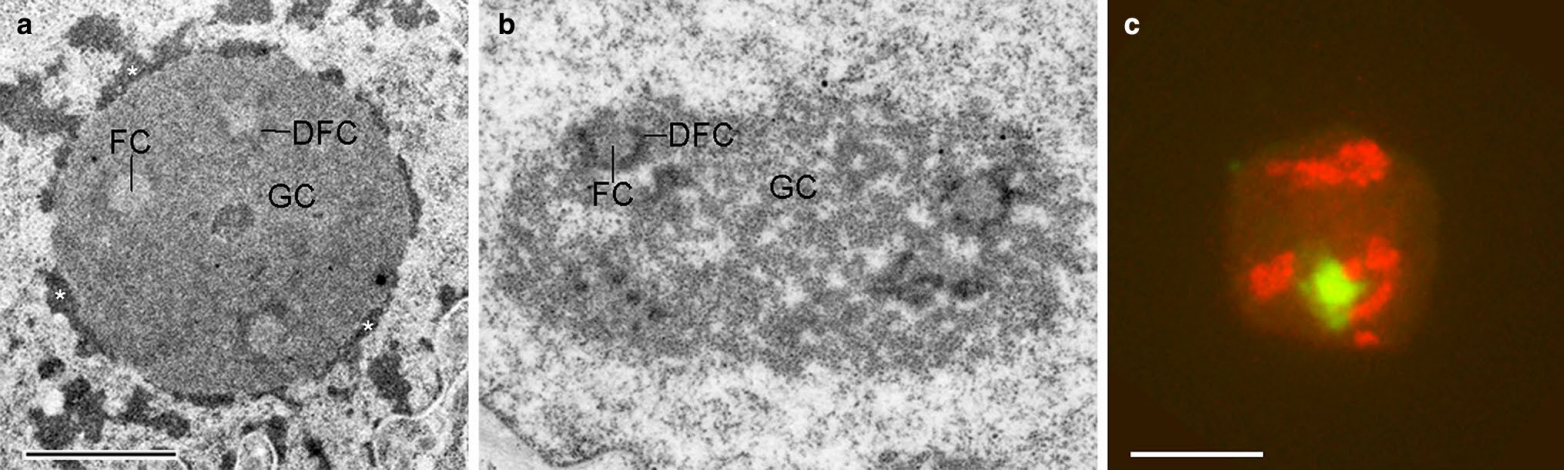
Electron micrographs of nucleoli (**a, b**) and a mouse testicular cell (**c**; spermatogonia). **a** Compact nucleolus with abundant perinucleolar heterochromatin (asterisks; N1–S1 rat hepatoma cell); bar 1 µm **b** nucleolus containing little perinucleolar heterochromatin but abundant intranucleolar chromatin (rat 9 G fibroblast). *FC* fibrillar center, *DFC* dense fibrillar component, *GC* granular component; same magnification as **a**. **c** FISH to detect rDNA (red) and X plus Y chromosomes (green; fusion of both sex chromosomes forms the sex vesicle). This image shows rDNA adjoining the sex vesicle as example for preferential spatial proximity of sex chromosomes to nucleoli in these cells; bar 10 µm

Nucleoli typically display a tri-partite organization as judged from ultrastructure. Roundish structures with electron-light, fibrillar interior, fibrillar centers (FCs) are surrounded by electron dense fibrillar stretches, dense fibrillar component (DFC), which may extend away from FCs. These fibrillar structures lie embedded in a large body of granular nature, granular component (GC), which dictates the overall shape of the nucleolus. In addition to these three components, patches of heterochromatin reside at the nucleolar periphery interspersed by euchromatin (Fig. 2a, b). From the perinucleolar heterochromatin, thin strands of condensed chromatin enter the nucleolar body (intranucleolar chromatin).

In the past, intense research efforts of several workgroups tried to correlate the structure of rDNA with the nucleolar components. Consensus has been reached in the finding that rDNA is located within the fibrillar parts of nucleoli, in the perinucleolar heterochromatin, and in intranucleolar stretches. In addition, ribosomal RNA was unequivocally localized to DFC and GC, and nascent transcripts identified by incorporated labeled precursor nucleotides were identified in the fibrillar parts of nucleoli. Together, these results demonstrated that, outside the nucleoli, there reside transcriptionally inactive rDNA repeats, whereas rRNA transcription occurs in the fibrillar areas. It should be noted that inactive rRNA genes might also be found in the intranucleolar chromatin as part of a loop of rDNA emanating out of perinucleolar (i.e., silenced) rDNA into the nucleolar interior. rRNA processing, which starts co-transcriptionally, is found in DFC and GC, in the latter of which assembly of rRNA with ribosomal proteins takes place. Unfortunately, although similar technical approaches were used, no consensus was reached on the exact localization of rDNA within the two fibrillar areas and as to where the site of transcription precisely is located (Thiry and Thiry-Blaise 1989; Wachtler et al. 1992). In one scenario, it is the FC where rDNA is residing, and initially, it was proposed that transcription takes place here. This view is supported by the existence of abundant Pol I molecules within FCs, which, in fact, is used as marker for this component (Scheer and Rose 1984). In this case, the DFC is the site of the early rRNA processing. Others showed that both genes and transcription can be localized in the DFC, whereas the FC functions as a source of Pol I molecules to be recruited to the DFC as it was shown that FCs contain predominately de-phosphorylated Pol I, representing the inactive state of the enzyme. In addition, a main constituent of DFCs is fibrillarin, a molecule implicated in maintenance of an open chromatin conformation required for transcription and in addition for rRNA elongation. Immunogold clusters after double labeling of nascent transcripts and detection of rDNA were found in the DFC (Mosgoeller et al. 1998). These clusters were found in a focal distribution within the DFC suggesting functional heterogeneity underlying the otherwise homogenous ultrastructural appearance of the DFC.At least some consensus may have been reached by placing the site of rRNA transcription to the interface between FCs and DFCs, while the exact localization of rRNA genes and intergenic spacer separating them has remained an open question until today.

Microscopic evaluation of fixed cells and averaging molecular methods like ChIP or the 3C variants may yield the misleading impression that the nucleolus is a rather stable organelle in the interphase nucleus. Fluorescence recovery after photobleaching (FRAP) revealed, however, that maintenance of the large-scale nucleolar structure is based on surprisingly dynamic behavior of its constituents. It was found that Pol I components are continuously exchanged at sites of rRNA transcription and thus only transiently localize to fibrillar components of nucleoli (Dundr et al. 2002). In vivo imaging of both Pol I components and fibrillarin indicates a pulsed transcription of rRNA (Hornacek et al. 2017), which resembles the transcription of Pol II-mediated genes.

The attempts to localize rDNA at ultrastructural level on ultra-thin sections (about 70 nm thick) made it difficult to evaluate the arrangement of entire rDNA repeats or even array segments thereof within the nucleolus. It should be kept in mind that an elongated Christmas tree is larger than the diameter of the average FC/DFC complex (4.4 µm versus approx. 1 µm). Clearly, rDNA fibers have to be present within nucleoli in a conformation providing a significant packaging while concomitantly ensuring massive transcriptional activity.

Combinatorial studies using histochemistry and immunogold methods, volumetric imaging, or ChIP, 3C data sets, and modeling led to the proposal of several models of the 3-D arrangement of rDNA within nucleoli. The DNA-cloud model proposes an inner core of DNA fibers located within FCs originating from condensed intranucleolar chromatin and further expanding in a radial fashion towards the periphery. These peripheral fine chromatin fibers overlap with DFCs where the transcriptionally active genes are located (Biggiogera et al. 2001). In another model derived from the simultaneous detection of Pol I molecules and nascent transcripts in 3D followed by modeling, Pol I molecules are grouped in the form of several coils, 60 nm in diameter, and located at the periphery of FCs (Cheutin et al. 2002). The core–helix model based primarily on high-resolution ChIP und 3C techniques shows preferential association between promoter, upstream region and the terminator region R3 located at the 3′ end of rDNA genes. According to this model, the association of these elements forms a core that is located inside FCs together with the IGS in general. The active genes would coil around this stable core and the authors suggest that these localize to the periphery of FCs (Denissov et al. 2011). The previous models support a functional morphology unique to Pol I transcription embedded in the nucleolar morphology. Recently, it was hypothesized that Pol I- and Pol II-dependent transcription share basic organizational principles despite different constituents that make up the transcription machineries (Weipoltshammer and Schöfer 2016).

## Nucleolar morphology and regulation of gene transcription

### rDNA and nucleosomes

Ultrastructural in situ detection of histones in nucleoli using acrolein fixation and silver nitrate staining demonstrated the presence of histones at the nucleolar periphery and in strands of intranucleolar DNA but not in fibrillar complexes (Derenzini et al. 1985). Many more insights were obtained by electron microscopy of spread preparations of rDNA. The Christmas tree images of Miller-spread preparations captured rRNA transcription in-flagranti, and demonstrated that more than 100 molecules of Pol I simultaneously are present on the rDNA template all involved in synthesis of nascent transcripts. Calculation of Pol I abundancy in HeLa cells confirmed the presence of about 100 Pol I molecules per gene and estimated about four transcribed rRNA genes per DFC/FC unit (Dundr et al. 2002; Haaf et al. 1991; Jackson et al. 1998).

In Miller-spread preparations under conditions retaining nucleosomes (Osheim et al. 2009), it was also noted that no histones were seen on rRNA genes, neither on the transcribed gene itself nor on the adjacent intergenic spacer sequences (Rattner et al. 1982; Scheer 1978). These observations led to the conclusion that the actively transcribed genes are devoid of histones and active rDNA is thus not present in nucleosomal conformation. This view was easily adopted as it was thought that a nucleosomal template would significantly reduce the speed of transcription, which was estimated to proceed at 95 nt/s (equals about 140 s for the entire 13.3 kb rRNA primary transcript). Upon transcriptional inactivation via actinomycin D (AMD), increasing histone occupancy was observed in both gene bodies and IGS in Miller spreads of amphibian oocytes (Scheer 1978). This shift from open to closed chromatin conformation was also reported in yeast under nutritial deprivation (and vice versa) using psoralen cross-linking approaches (Dammann et al. 1993; Johnson et al. 2013). Interestingly, Miller-spread preparation in yeast revealed that a minor fraction of active rRNA genes retained some nucleosomes, whereas the vast majority of ribosomal genes were without nucleosomes as is the IGS (Johnson et al. 2013). Albert et al. (2012) could identify irregularly spaced nucleosomes within stretches of inactive yeast rRNA genes.

Psoralen- and formaldehyde-cross-linking (Conconi et al. 1989; Stancheva et al. 1997) and later chromatin endogenous cleavage (ChEC) using micrococcus nuclease digestion experiments (Merz et al. 2008) permitted to discriminate between nucleosomal and non-nucleosomal chromatin. Using these approaches, the existence of two populations of rDNA, one with (closed configuration) and one without (open configuration) nucleosomal organization, has been demonstrated in yeast and mammalian cells. It was, hence, concluded that the latter represents actively transcribed rDNA, whereas the former fraction contains condensed, transcriptionally silent rDNA repeats. This raised the question by which factors the histone-free organization of rRNA genes is maintained. Merz et al. (2008) identified the major nucleolar protein, Hmo1 (high mobility group protein 1) to accomplish this task *Saccharomyces cerevisiae*. The mammalian Hmo1 orthologue upstream-binding factor (UBF) is long known to be an essential factor for rRNA transcription and has recently been identified as factor responsible for defining the chromatin state of actively transcribed mammalian rRNA genes (Herdman et al. 2017). These authors also show that UBF delimits rRNA genes, is largely absent from IGS regions and is required for targeting the preinitiation complex to promoters, which precedes the start of transcription. While UBF seems to be responsible for maintenance of histone-free regions, it is, to date, not clear, if UBF is sufficient to establish the histone-free state. In yeast, the establishment of histone-free regions can be induced by Pol I activity (Wittner et al. 2011). UBF has been identified as essential prerequisite for recruitment of the Pol I transcription machinery in mammals (Grob et al. 2014).

The concept of histone-depleted active rRNA genes was challenged by findings using chromatin immunoprecipitation (ChIP), which led to the conclusion that significant amounts of histones are present on active rRNA genes not impeding Pol I transcription in yeast (Jones et al. 2007) and in mammalian cells (Foltankova et al. 2013). Interestingly, in protozoa, it was recently reported that nucleosomal organization exists within the coding region and the IGS, whereas the promoter is free of histones (Vizuet-de-Rueda et al. 2016), which might point to evolutionary differences in nucleolar chromatin organization. In a complementary approach, decreased histone H3 levels in yeast cells resulted in strongly reduced rRNA synthesis and the authors concluded that histone association is functionally important for rRNA transcription (Tongaonkar et al. 2005).

In a recent paper using ChIP-Seq approach, it was found that the rRNA gene body and the IGS may be devoid of histones in actively transcribing genes, whereas the enhancer boundary complex within the IGS upstream of the enhancer repeat elements is the only site of active histone modification (Herdman et al. 2017) and thus of nucleosomal organization. An integrative genomic analysis comparing rDNA chromatin to non-ribosomal genome elements confirmed that the coding region of human rDNA is histone-depleted, whereas the IGS and gene promoters display histone modifications and thus histones (Zentner et al. 2011). In yeast, nucleosomal organization was found on rDNA promoters (Johnson et al. 2013). Chromatin remodeling activities lead to displacement of promoter-bound nucleosomes 22 bp upstream to allow access of the transcription initiation machinery in mammals (Li et al. 2006), much like a switch that has been turned “on”. Chromatin remodeling occurs prior to rRNA gene activation, and thus, the authors argue that the repressed state is the default chromatin organization of the rDNA and gene activation requires ATP-dependent chromatin remodeling activities (Felle et al. 2010). Apart from active and silenced rRNA genes, a third population of rRNA genes was discovered that are transcriptionally inactive yet in an open chromatin conformation. These genes are thought to be present in a poised state (Xie et al. 2012). The promoter of these rRNA genes is unmethylated, and components of the preinitiation complex of the transcription machinery are associated with the promoter, while the nucleosomes are in an “off”-position refractory to transcription initiation. The chromatin displays bivalent histone modifications signifying both euchromatin (H3K4me3) and heterochromatin (H3K27me3) signatures (Xie et al. 2012).

Taking together, there exist three populations of rRNA genes defined by their epigenetic signatures: active, silenced, and poised genes.

### rDNA and histones

It had been suggested that, if nucleosomes are present at all in actively transcribed genes, these exist in a non-canonical nucleosome particle composition [for a review on these, see Lavelle and Prunell (2007)]. An alternate, extended nucleosome core particle composition had been proposed to exist in rDNA, referred to as lexosome (Judelson and Vogt 1982; Prior et al. 1980), yet the existence could not be confirmed in in vitro assays (Protacio and Widom 1996).

The non-canonical histone H3 variant H3.3 was associated with ongoing Pol II-mediated transcription (Ahmad and Henikoff 2002). Its nuclear distribution pattern coincides with markers for active chromatin in human cells (Goldberg et al. 2010; Snyers et al. 2014), but also repetitive sequences such as telomeres and centromeres contain H3.3 (Boyarchuk et al. 2011; Wong et al. 2010). The high transcription rate and the repetitive nature suggest the presence of H3.3 in rDNA. Using structured illumination imaging (SIM), no apparent H3.3 signal could be seen within nucleoli such as was the case with canonical histones (Snyers et al. 2014). However, in plants, H3.3 presence in nucleoli has clearly been demonstrated (Shi et al. 2011), and very recently, H3.3 occupancy was demonstrated by ChIP analysis in mammalian rRNA genes (Udugama et al. 2018). The authors relate H3.3 occupancy to rDNA silencing rather than transcriptional activity.

The H2A variant macroH2A1, which is normally not detected in rDNA, was found to be recruited to rDNA upon inhibition of Pol I transcription or nucleolin knockdown in mammalian cells (Cong et al. 2014). The phosphoprotein nucleolin is required for Pol I transcription and is thought to bind histones, thereby facilitating transcription (Rickards et al. 2007). Novel evidence indicates that nucleolin might be required for controlling the transcriptional ON/OFF states of rDNA chromatin in both mammals and plants (Durut and Saez-Vasquez 2015).

Another histone H2A variant, H2A.Z is enriched at promoter regions of euchromatin (Meneghini et al. 2003). It was suggested that H2A.Z incorporation into rDNA chromatin is maintained by the actin-related protein ARP6 under high glucose conditions, which is part of the Snf-2-related CREB-binding protein activator protein (SRCAP) chromatin remodeling complex (Kitamura et al. 2015). In another study, it was shown that H2A.Z is present in the IGS (Hamdane et al. 2014). Interestingly, in Pol II-mediated transcription, alternative nucleosome core particles containing both the histone variants H3.3 and H2A.Z have been identified that are enriched at “nucleosome-free regions” of active promoters. The authors report that the particles are unstable and that this instability might facilitate the accessibility of the transcription machinery to rDNA sequences (Jin et al. 2009). It remains to be seen, if these two histones associate in nucleoli and bind to actively transcribed ribosomal DNA. Histone H1 is enriched in inactive rRNA genes (Hamdane et al. 2014). Phosphorylated histone H1 variants H1.2 and H1.4 have been identified in human rDNA and, surprisingly, their association with rDNA was found to correlate positively with transcriptional activity (Hamdane et al. 2014; Zheng et al. 2010).

### rDNA and epigenetic regulation

Epigenetic mechanisms play important roles in transcription control of Pol II genes and are crucially important for rRNA transcription control (McStay and Grummt 2008).

DNA methylation is an important epigenetic factor in gene transcription control generally correlated with gene silencing [recently reviewed in Lyko (2018)]. Psoralen cross-linking studies revealed methylation of the rDNA promoter region and, to a lesser extent enhancer regions and is found enriched in inactive rDNA copies (Stancheva et al. 1997). DNMT1 is the DNA methyltransferase responsible for the maintenance of genomic CpG patterns and loss of DNMT1 does not only lead to erasure of methylation in rDNA but, in particular, to disorganization of the nucleolar structure (Espada et al. 2007). In a recent EM study, methylated DNA has been detected at the nucleolar periphery but also on intranucleolar DNA strands (Masiello and Biggiogera 2017) suggesting the presence of transcriptionally silent rDNA. In contrast, the authors found no DNA methylation signal in the fibrillar compartments which is in agreement with the occurrence of actively transcribed genes there. Loss of DNMT1 has, indeed, been shown to lead to transcriptional activation of previously silent genes, which was correlated with ectopic Pol II transcription on rRNA genes and accompanied by accumulation of primary rRNA transcripts. This led to the suggestion that rDNA methylation prevents Pol II transcription (Gagnon-Kugler et al. 2009). More recently, it was shown that DNMT1 occupancy at rRNA genes is mediated by the NAD+-dependent histone deacetylase enzyme SIRT7 (Ianni et al. 2017). Loss of SIRT7 led to reduced DNMT1 presence on rRNA genes, to nucleolar fragmentation and loss of rRNA genes thus linking rDNA methylation with rDNA array stability (Paredes et al. 2018). The de-novo DNA methyltransferase DNMT3b has been implicated in methylation of CpG residues in the promoter, thereby impairing binding of UBF and, consequently, counteracting the assembly of the transcription complex. DNMT3b recognizes specific promoters by the presence of R-loops formed by Pol I-mediated IGS transcripts (Bierhoff et al. 2014a).

Histone modifications represent another layer of transcriptional regulation. Numerous examples for histone remodeling have been identified in rDNA imparting silencing and activating marks. The inactivation of active rRNA genes was shown to depend on the nucleolar remodeling complex NoRC in mammals (Strohner et al. 2001) that interacts with the transcription terminator factor (TTF-I). NoRC leads to displacement of core histone particles, thereby preventing binding of the Pol I transcription machinery. NoRC associates with both DNA methyltransferases and histone de-acetylases (DNMT1, DNMT3b; HDAC1, SIRT1), which leads to a stable and inherited repressed state of rDNA (Li et al. 2006; Santoro et al. 2002). Furthermore, silencing relies on the energy-dependent nucleolar silencing complex [eNoSC; (Murayama et al. 2008)], which contains SIRT1, nucleolar protein nucleomethylin (NML), and the histone methyl transferase SUV39H1, an enzyme responsible for methylation of lysine 9 of H3 histones (Peters et al. 2001). It was further shown that eNoSC binds H3K9me2 in rDNA and also binds to rRNA and thus links transcript elongation to transcriptional activity in a nutritient-dependent way that was supposed to be reversible upon nutrient availability in human cells (Yang et al. 2013). Another complex that silences rDNA is the NuRD complex that contains, amongst many other components class I histone deacetylase (HDACs) 1 and 2 (Zhang et al. 1999). Interestingly, the activation and binding to rDNA promoters leads to bivalent histone modification markers representative for both inactive and active chromatin such as H3K27me3 and H3K4me3, respectively (Xie et al. 2012). It has thus been proposed that NuRD-bound rDNA is accessible for Pol I transcription machinery but remains transcriptionally inactive. In this state, the promoter is occupied by the preinitiation complex without Pol I. Hence, it was concluded that this represents a poised state, which is transcriptionally silent but remains permissive for transcriptional activation. Recently, the helicase SNF2 histone linker PHD RING helicase (SHPRH), a functional homolog of yeast Rad5, has been suggested to be responsible for sensing of poised rDNA promoters and for localizing Pol I to these promoters via interaction with H3K4me and H3K4me2 (Lee et al. 2017, 2018). Furthermore, the authors identified the mammalian target of rapamycin (mTOR) pathway implicated in this process, thus linking gene activation with environmental cues.

Lysine-specific de-methylases (KDMs) of the JmjC family were found to fulfill dual roles in rDNA transcription control. KDM2A and KDM2B induce silencing, whereas KDM4B removes silencing marks. KDM2A de-methylates H3K36 at the rDNA promoter (Tanaka et al. 2010) and KDM2B de-methylates H3K4, which dislocates UBF from the rRNA gene body (Frescas et al. 2007). KDM4B de-methylates H3K9, and is found in promoters and rRNA gene bodies of cancer cells (Bartova et al. 2011) suggesting an activating role in rRNA transcription (Zhou et al. 2009). KDM2B knockdown in transformed and non-transformed mammary gland cells increases rRNA transcription and is associated with increased cancerous behavior (Galbiati et al. 2017). Another member of the JmjC family, PHD finger protein (PHD8), removes repressive methylation marks from H3K9me2 on promoters, thereby stimulating rRNA transcription (Feng et al. 2010; Zhu et al. 2010).

Enzymes involved in histone acetylation are another group of important regulators of rDNA activity (Hirschler-Laszkiewicz et al. 2001). Histone acetyl transferases (HATs) have been shown to fulfill divergent roles. KAT5 (also known as Tip60) overexpression is associated with reduced rDNA promoter activity (Koiwai et al. 2011). KAT8 is required for H4K16 acetylation at rDNA promoters, which implies an activating role. However, KAT8 is also required for nucleolar NoRC complex association during S phase, suggesting different roles of this enzyme during the cell cycle. Acetylation of lysine 14 of histone H3 (H3K14ac) has been reported to be important for rDNA silencing (Xu et al. 2016), whereas acetylation of H3K56 triggered by the TOR pathway creates chromatin permissive for Pol I transcription (Chen et al. 2012). Furthermore, HATs were implicated in conversion of poised to active rDNA state. Histone acetyltransferase P300/CBP-associated factor (PCAF) was shown to become recruited to promoters by the histone remodeling factor Cockayne syndrome B (CSB) where it acetylates histones H3 and H4, which is required for the assembly of the Pol I initiation complex (Shen et al. 2013).

Notably, a nucleolus-specific histone modification pathway has been identified leading to glutamine methylation of histone H2A. Here, the prominent nucleolar protein fibrillarin was identified as the responsible methyl transferase enzyme (Tessarz et al. 2014).

The histone de-acetylases (HDACs) are generally associated with transcriptional silencing. HDAC1 and HDAC2 interact with NoRC and facilitate the silencing of rDNA (Zhou et al. 2002). The NAD+/NADH-dependent class III HDACs, sirtuins, transmit environmental stress to rRNA transcription. SIRT1 de-acetylates H3K9 and H4K16 leading to rDNA silencing (Murayama et al. 2008). SIRT7 is predominately localized in nucleoli and associates with DNMT1, SIRT1, UBF, histones, and Pol I (Ford et al. 2006; Grob et al. 2009; Ianni et al. 2017; Tsai et al. 2012). It was suggested that SIRT7 acts as an activator of rRNA transcription (Dass et al. 2016; Ford et al. 2006; Murayama et al. 2008), although another study demonstrated that SIRT7 is active in the establishment and maintenance of silent rDNA (Ianni et al. 2017). In the latter study, reduction of SIRT7 leads to hypomethylation of rDNA and histone hyperacetylation but also to nucleolar fragmentation, suggesting a role in nucleolar structure maintenance. Nucleolar localization of SIRT7 is tissue- and age-dependent (Kiran et al. 2013), and is shown to protect against senescence in normal cells (Paredes et al. 2018).

### Nucleolar chromatin organization: structural determinants

Condensins and insulator factor CTCF (CCCTC-binding factor) seem to have inverse roles on rRNA transcription. The former acts as a negative regulator, whereas the latter promotes transcription in mammalian cells (Huang et al. 2013). In contrast, in *Drosophila*, CTCF represses transcription. Interestingly, CTCF seems to be enriched in those transposable elements (Guerrero and Maggert 2011) that are interspersed within rDNA arrays of many non-vertebrate eukaryotic taxa (Zhou et al. 2013). CTCF has been detected in DFC/GC of rat neurons, whereas, in cycling cells, the expression pattern was rather nuclear than nucleolar (Torrano et al. 2006). It was also established that CTCF interacts with UBF (Sanij and Hannan 2009; van de Nobelen et al. 2010), and knockdown of CTCF leads to nucleolar disintegration and defects in DFC morphology (Hernandez–Hernandez et al. 2012). Cohesin has been implicated in loop formation and mutations in the genes coding for cohesin and its regulatory factors lead to nucleolar disruption (Harris et al. 2014; Xu et al. 2014). ChIP-Seq experiments revealed that CTCF and cohesin bind to the unique enhancer boundary complex, which remains stable after gene inactivation and it was thus suggested that the boundary complex is involved in the maintenance of a poised state of rDNA (Herdman et al. 2017). In a very recent Hi-C attempt to generate a long-range contact map of rDNA, a significantly higher percentage of 47S rDNA contacts with CTCF-binding sites was identified in rDNA sequences carrying repressive marks than in those with active signatures (Yu and Lemos 2018).

Interestingly, a potent inducer of rRNA transcription is c-Myc, which associates with rRNA gene looping structures in cycling cells (Shiue et al. 2009). Moreover, the authors demonstrated that the c-Myc-induced loops of active rDNA are attached to the nucleolar matrix via the IGS region, whereas inactive rRNA genes are not anchored at the matrix (Shiue et al. 2014). A differential transcription-dependent anchoring of rRNA genes has been reported previously using a nucleoskeleton extraction protocol (Weipoltshammer et al. 1999). However, here, the rDNA region attached to the nucleoskeleton was rather located within the gene body (Weipoltshammer et al. 1996b). In another earlier study of our group, applying the halo extraction method, long-range rDNA loops could be directly visualized emanating from NORs (Schöfer et al. 1998). More recently and in contrast to the previous reports, it was shown that the rDNA silencing NoRC complex component Tip5 is involved in association of rRNA genes with the nucleolus by targeting the nuclear matrix and that the rDNA–matrix association is increased under the serum starvation conditions (Zillner et al. 2013).

An additional organizing principle that has to be taken into account for formation and maintenance of nucleoli are biophysical properties inherent to major nucleolar constituents, which have the potential of nucleolar self-assembly. Biophysical processes studied in this respect so far are molecular crowding in the nucleus [reviewed in Hancock (2014)] and liquid–liquid phase separation effects (Berry et al. 2015).

### RNA, epigenetic regulation, and nucleolar structure

Another layer of epigenetic regulation of rRNA transcription is non-coding RNA (ncRNA).

Promoter elements structurally similar to rRNA gene promoters and transcribed by Pol I were identified in the IGS previously. One of these transcripts derived from upstream of the rDNA promoter is involved in rDNA silencing by binding to NoRC and targeting the complex to the promoter (pRNA; Mayer et al. 2006, 2008). Two other, long non-coding RNAs (lncRNAs) have been detected in the IGS that are activated by environmental cues and cause reversible re-organization of the nucleolar structure (detention center) under stress conditions (Jacob et al. 2013). Surprisingly, also Pol II-mediated transcription of rRNA genes was identified albeit at low levels. Antisense transcripts derived from regions spanning rDNA promoter and gene body (PAPAS; promoter and prerRNA antisense) were identified and their presence anti-correlated with rRNA levels suggesting a role in rDNA silencing (Bierhoff et al. 2014a, b). This fits well with the earlier work, demonstrating, in yeast, that H3K4 methylation mediated by the methyltransferase Set1 is required for repression of Pol II transcription within rDNA (Briggs et al. 2001). In addition to these transcripts that are “endogenous” to nucleoli, i.e., transcribed from rDNA, RNAs from non-nucleolar templates have been detected within nucleoli such as Pol II-transcribed transposable elements (Alu) RNAs, which may have a role in maintenance of nucleolar structure via interaction with nucleolin (Caudron-Herger et al. 2015).

## Nucleolus, perinucleolar chromatin, and organization of nuclear chromatin

The perinucleolar chromatin representing the chromatin touching the nucleolar periphery is important for structure and function of the nucleolus itself as well as for the organization of the genome (Manuelidis and Borden 1988). The perinucleolar chromatin consists of both eu- and heterochromatin stretches.

### Silent ribosomal genes (47S rDNA)

The association of NORs with nucleoli is independent of Pol I transcription as has been shown in mouse-human hybrid cells where the inactive human NORs abut the murine nucleolus (Sullivan et al. 2001). The number of inactive NORs located at the nucleolar periphery varies in cycling cells and is inversely correlated with transcriptional activity of the nucleolus. The extra-nucleolar position may be necessary for maintaining these rDNA arrays in an inactive chromatin conformation. Very recently, the first long-range interaction map of rDNA was published demonstrating that rDNA–rDNA contacts preferentially occur in closed, repressed, and late replicating domains (Yu and Lemos 2018). It can be speculated that these contacts preferentially refer to the extra-nucleolar silent rDNA clusters.

### 5S rDNA

In yeast, it was shown that an ectopic ribosomal DNA repeat silences the chromosomal region in which it is integrated by relocalizing it to the perinucleolar position (Jakociunas et al. 2013), suggesting that the nucleolar periphery represents a domain repressive for transcription. The previous studies demonstrated the preferential association of Pol III-transcribed genes with nucleoli in particular of 5S rRNA genes (Matera et al. 1995). Like 47S rRNA genes, 5S rDNA is also present in a tandem array conformation and 5S rRNA transcription is involved in the regulation of 47S rRNA transcription. It has been hypothesized that the perinucleolar position of 5S rDNA facilitates ribosome biogenesis (Haeusler and Engelke 2006). On the other hand, the perinucleolar position of mouse 5S rDNA is anti-correlated with transcriptional activity confirming the repressive nature of the perinucleolar area (Fedoriw et al. 2012). It should be noted that, in Matera et al. (1995), the 5S rDNA array was reported to be localized near to the nucleolus but not being part of the perinucleolar compartment. Along this line, two recent papers using Hi-C found that 5S and 47S rDNA do not physically interact (Yu and Lemos 2016, 2018). Taking these findings together, it is tempting to speculate that active 5S rRNA genes reside near nucleoli in euchromatin environment outside the repressive perinucleolar compartment, while they become recruited to the latter during transcriptional silencing.

### NOR-bearing chromosomes

NORs are embedded in chromosome territories and have to physically convene to form a nucleolus. In extreme cases such as in neurons, most NORs fuse into one cluster of perinucleolar NOR-chromatin. Human NORs are located at the p arms of acrocentric chromosomes, but little is known about the flanking regions of the NORs. Recently, NOR-junction regions could be isolated and in particular the NOR-flanking region close to telomeres (distal junction) preferentially associate with nucleoli. Interestingly, although located predominately in the perinucleolar heterochromatin, these sequences display genomic architecture similar to euchromatin and are transcribed (Floutsakou et al. 2013). It could further be shown that ectopically located distal junction sequences formed association with nucleoli (Floutsakou et al. 2013), while ectopic rDNA arrays without distal junction sequences did not associate with nucleoli (Grob et al. 2014).While the mechanisms by which NORs convene are not fully understood Pol I transcription seem to be dispensable for NOR fusion (Dousset et al. 2000). Distal and proximal NOR-flanking sequences have been found implicated in maintaining nucleolar integrity and, possibly, in NOR fusion (Floutsakou et al. 2013) but not in the formation of nucleoli (Grob et al. 2014).

Proximal to the proximal junction lies centromeric and peri-centromeric regions. Centromeres frequently associate with nucleoli (Ochs and Press 1992). Most prominently, in Purkinje neurons, the centromeres fuse and associate with nucleoli building a large block of perinucleolar heterochromatin (Manuelidis 1984). Mutating the probable *Drosophila* homolog of the major nucleolar protein nucleophosmin (NPM, B23), nucleoplasmin-like protein led to centromere re-location from nucleoli and was thus suggested to be implicated in nucleolar tethering in concert with CTCF (Padeken and Heun 2014).

Using whole-chromosome FISH for the human NOR-bearing acrocentric chromosomes, it was found that the entire chromosomes showed a preference to be near nucleoli (Kalmarova et al. 2007).

### Sex chromosomes

Association of sex chromosomes with the nucleolus are long known (Gates 1939). For example, the nucleolus often associates with the sex vesicle during mouse spermatogenesis (Fig. 2c). Using FISH, the frequent association of sex chromosomes and nucleoli (and the nuclear lamina) could be confirmed in interphase of many human cell types [e.g., Manuelidis and Borden (1988); Weipoltshammer et al. (1996a)]. Consequently, the inactive X-chromosome (Xi) has also early been noted as dark-staining dot close to nucleoli in female cat neurons as “nucleolar satellites” (Barr and Bertram 1949) before it was identified as Xi. The perinucleolar positioning has been hypothesized to contribute to the silencing of Xi (Zhang et al. 2007). Nucleolar association of Xi was confirmed by sequencing approaches (Dillinger et al. 2017). In addition, the Y chromosome could be identified as perinucleolar chromatin constituent (van Koningsbruggen et al. 2010).

### The perinucleolar compartment (PNC)

The PNC occurs as a crescent-like structure at the surface of cancer cells and is related to malignancy. PNCs contain many RNA-binding factors and RNAs, in particular Pol III-derived transcripts, although PNCs are not the site of their transcription. PNCs remain associated with nucleoli throughout the cell cycle, but neither the factors responsible for this association nor its molecular functions are known yet (Wen et al. 2013).

### Nucleolus-associated domains (NADs) and in between (iNADs)

In 2010, two independent papers were almost simultaneously published where genomic elements were sequenced after isolation of human nucleoli, which led to the identification of specific chromatin areas termed NADs [nucleolar-associated (chromatin) domains; (Nemeth et al. 2010; van Koningsbruggen et al. 2010)]. Both papers provide for the first time a whole-genome view on perinucleolar chromatin. NADs are 0.1–10 Mb in size, relatively gene-poor, and enriched in satellite repeats. Amongst genes found in NADs were those for transcription factors, immunoglobulin genes, and olfactory receptor genes. A recent paper using Hi-C deep sequencing of cycling and senescent fibroblasts confirmed the mainly heterochromatic nature of NADs and that they cover about 38% of the annotated genome in cycling fibroblasts (Dillinger et al. 2017).An intriguing question is to which extent NADs contain specific chromatin domains. There exists some disagreement as to the correlation of the perinucleolar heterochromatic region with the other prominent nuclear heterochromatin compartment, the nuclear lamina to which lamina-associated chromatin domains (LADs) are attached to Guelen et al. (2008).

The dynamics of LADs were investigated using the DNA tracer technique DamID where it was shown that a large percentage of LADs do not inherit their position at the nuclear periphery through the cell cycle (Kind et al. 2013; Ragoczy et al. 2014), and some former LADs re-positioned to the nucleolar periphery (Ragoczy et al. 2014). These findings argue for a high degree of interchangeability between NAD and LAD sequences. On the other hand, a recent paper showed that NADs and LADs largely overlap in human fibroblasts; however, about one-third is non-overlapping (Dillinger et al. 2017) arguing for some heterochromatin domains with perinucleolar specificity. The authors also separately analyzed NADs and regions between NADs, termed inter-NADs (iNADs), and found that the latter are more gene-rich in nature than NADs. This result fits nicely to the nucleolar morphology where it is long known that the perinucleolar chromatin is of both eu- and heterochromatin nature (see Fig. 2a).

### Positioning of the perinucleolar chromatin

The interchangeability of NADs with LADs is contrasted by differences in morphology and composition of the two nuclear compartments. In vertebrate cells, the nuclear periphery is occupied by a rim of heterochromatin constituting the LADs, which is only perforated by nuclear pores (NPs) and their adjacent chromatin. A complex meshwork made of intermediate filaments, the nuclear lamina, mediates between the chromatin and the nuclear membrane, and consists of a well-defined set of interacting factors that are enriched at the nuclear periphery. In vertebrates, the nuclear periphery seems to be primarily repressive for transcription with the exception of the active chromatin surrounding the NPs (Fiserova et al. 2017). The nucleolar periphery in contrast is occupied by heterochromatin interspersed with larger euchromatin volumes, suggesting that the nucleolar periphery is less repressive than the nuclear periphery. Although some constituents of the nuclear lamina can be found within the nucleoplasm (Naetar et al. 2017), it appears that they are not enriched around nucleoli in the way that they are at the nuclear periphery. Interestingly, a recent study reported lamin B association with nucleoli and, more specifically with nucleolin. Rescue experiments of mutant lamin B suggest independent roles for lamin B2 at the nucleolus and nuclear envelope (Sen Gupta and Sengupta 2017). The histone H2A variant macroH2A occupies the Xi that is found in NADs. Moreover, macroH2A binds lamin B (Fu et al. 2015) and disruption of this histone variant alters nucleolar morphology, which may point to an important role for NAD heterochromatin organization (Douet et al. 2017).

CTCF has been implicated in tethering insulator regions of genomic elements to the nucleolar periphery mediated by the nucleolar protein nucleophosmin [NLP1, B23; (Yusufzai et al. 2004)]. NLP1 depletion leads to deformed nucleoli and a striking rearrangement of perinucleolar heterochromatin (Holmberg Olausson et al. 2014). NLP1 binds also centromere protein A (CENP-A; Foltz et al. 2006) suggesting a function in centromere tethering at the nucleolar surface. Indeed, in plants it has been shown that nucleolin is involved in both regulation of rRNA transcription and genome organization (Picart and Pontvianne 2017).

Other proteins involved in NAD positioning include p150 (Smith et al. 2014) and Ki-67 (Booth et al. 2014). Interestingly, Fedoriw et al. (2012) could also demonstrate that a 119 bp 5S rDNA sequence promotes nucleolar tethering. As this sequence frequently occurs in the genome, it might serve as more general mechanism to anchor genomic elements at the nucleolar periphery. Apart from proteins also RNAs, in particular, long non-coding RNAs (lncRNAs) were implicated such as Firre that was found to be involved in the Xi anchoring at nucleoli and is itself regulated by CTCF (Yang et al. 2015).

## Outlook/concluding remarks

**Fig. 3:**
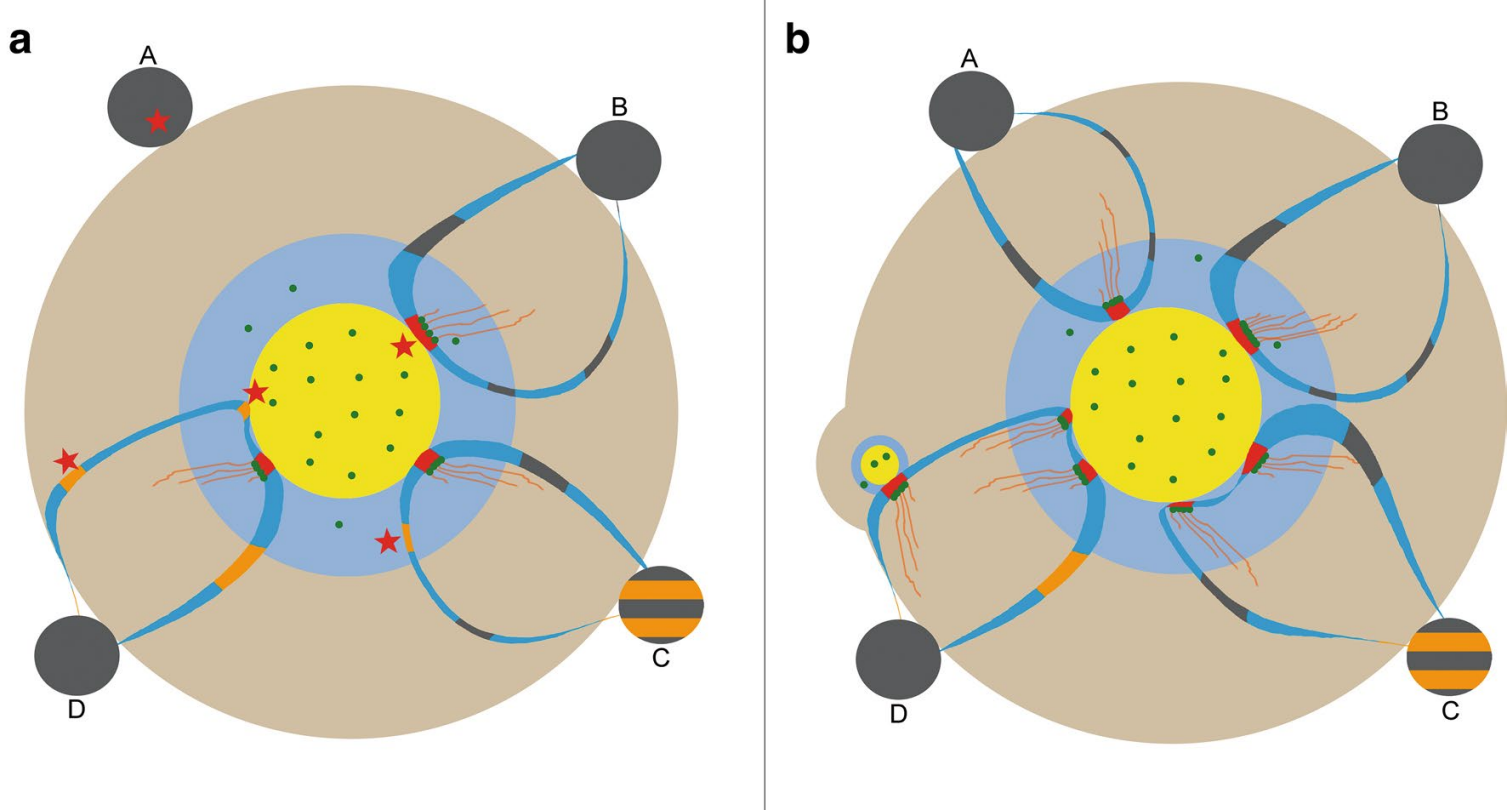
Scheme depicting known and hypothesized nucleolar structure function relation of different rDNA chromatin states under differing transcriptional demands. **a** Low demand for ribosomes: silent NORs (black) reside at the nucleolar surface, no engagement in transcription (A); loops composed of mostly silent rRNA genes (black) protrude into nucleoli as intranucleolar chromatin stretches; actively transcribed rRNA genes (red; Christmas trees) are present in the fibrillar complexes; blue—IGS (B). The localization of poised genes (orange) is presently not known; they may be interspersed with silent rDNA in transcriptionally silent NORs (C) or being part of the intranucleolar chromatin stretches (C, D). In **b**, some theoretical possibilities are depicted how recruitment of genes (marked with red asterisk in **a** upon increasing demand for ribosome synthesis can be envisaged: formerly inactive NOR-rDNA forms a loop into the nucleolar interior until they contact a fibrillar complex (A); in yeast, occupancy of rRNA genes with Pol I molecules increases (B); activation of poised genes by recruitment from NORs (not depicted) or of poised genes located near the existing FC/DFC domains (C); activation of poised genes located within intranucleolar chromatin may lead to formation of new FC/DFC complexes or re-activation of previously active genes that retained their position in a fibrillar complex until the epigenetic switch is turned on again (D). Yellow—fibrillar center, blue—dense fibrillar component, pale brown—granular component; green dots—Pol I molecules, and red–orange fibers—nascent rRNA transcripts

Ribosomal genes and the nucleolus faced renewed interest in recent years largely owing to the development of molecular and bioinformatics tools to address the repetitive nature of rDNA arrays. Microscopy has also seen great improvements to increase optical resolution. Particularly noteworthy is the development of the various super-resolution methods both on the instrumentation side (e.g., SIM, STORM, STED, etc.) and in preparation improvements [expansion microscopy; (Chen et al. 2015)]. These methods offer now sufficient resolution for the investigation of the functional architecture of nucleoli and it seems likely that the number of nucleolus papers exploiting the potential of these technologies will rise in the near future. Indeed, there are still open questions to address in a structure–function context. One such question relates to the dynamics of gene silencing and activation, respectively, depending on environmental cues. Particularly little is known on the nucleolar location and dynamics of poised rRNA genes, so that, in the meantime, one can only resort to speculations as to their structure–function relation (Fig. 3). Further questions relate, e.g., to the chromatin nature of the intranucleolar rDNA stretches, in general, or to the IGS and its histone occupancy and precise nucleolar position. Certainly, electron microscopy will continue to be a valuable tool and correlative light and electron microscopy (CLEM), especially when combined with super-resolution microscopy may become a promising avenue to gain further insights.

Another very interesting focus is the heterogeneity of the rDNA array, both in terms of genetic variability and in epiallelic composition. Such studies have already revived the interest of pathologists in rDNA and nucleoli and will certainly continue to do so, particularly in oncology. Nucleoli recently became therapeutic targets for small anti-cancer drugs specifically inhibiting the Pol I machinery (Drygin et al. 2011). Finally, it is an interesting clinical perspective to integrate defects in rDNA composition and rRNA synthesis into a wider view of a heterogeneous group of disorders that result from ribosome dysfunctions called ribosomopathies (Mills and Green 2017).

Recent progress made in deciphering the spatio-temporal functional architecture of the nucleolus will enhance our understanding of fundamental cellular processes such as metabolic adaptation to environmental conditions, senescence, and aging leading to the improved diagnosis and treatment of diseases.
